# Association of environmental, demographic and clinical parameters with physical activity in children with asthma

**DOI:** 10.1038/s41598-025-87426-5

**Published:** 2025-01-22

**Authors:** Jane Berrill, Peter James, Antonis Michanikou, Emmanouil Galanakis, Eleni Michaelidou, Paraskevi Kinni, Nikos Kalivitis, Giorgos Kouvarakis, Emily Vasiliadou, Chrysanthos Savvides, Filippos Tymvios, Petros Koutrakis, Panayiotis K. Yiallouros, Panayiotis Kouis

**Affiliations:** 1 Department of Environmental Health, Harvard TH Chan School of Public Health, Harvard University, Boston, USA; 2 Medical School, University of Cyprus, Nicosia, Cyprus; 3 Medical School, University of Crete, Heraklion, Greece; 4 Department of Chemistry, University of Crete, Heraklion, Greece; 5 Air Quality Section, Department of Labour Inspection, Ministry of Labour and Social Insurance, Nicosia, Cyprus; 6 Department of Meteorology, Rural Development and Environment, Ministry of Agriculture, Nicosia, Cyprus; 7 Shacolas Educational Centre for Clinical Medicine, 215/6 Palaios Dromos Lefkosias Lemesou, Aglantzia, Nicosia, 2029 Cyprus

**Keywords:** Childhood asthma, Physical activity, Pedometer, Weather, Air pollution, Paediatric research, Epidemiology

## Abstract

Personal characteristics, unfavorable weather conditions and air pollution have been linked with reduced physical activity in children. However, among children with asthma the effects of these parameters remain unclear. This study objectively quantified the physical activity of children with asthma and evaluated its association with environmental, personal, and clinical parameters. Participants of the prospective LIFE-MEDEA asthma study wore the EMRACE™ smartwatch daily for continuous monitoring of physical activity and acquisition of global positioning system data. Daily physical activity, personal and clinical data were combined with daily temperature, precipitation, and air pollution levels in adjusted mixed effect regression models to examine the relationship between physical activity and the examined parameters. For a follow-up period of 4 months, 186 children with asthma demonstrated a decrease of 796 steps (95% CI: -1080, -512) on days with precipitation compared to non-precipitation days and a decrease of 96 steps (95% CI: -182, -9) for every 10 µg/m^3^ increase in PM_10_. The relationship between temperature and daily steps was characterized by an inverted U-shape. There was also evidence that gender and age-adjusted BMI z-score were negatively associated with daily steps. These results can further inform the design of physical activity interventions targeting children with asthma.

**Supplementary Information:**

The online version contains supplementary material available at 10.1038/s41598-025-87426-5.

## Introduction

Asthma is the most common chronic condition of childhood^1^ and despite the use of existing medications, it may impact physical development, school attendance, physical activity, and overall quality of life of children^2–5^. The relationship between asthma and physical activity is complex. Intense physical activity may trigger asthma symptoms for 40–90% of patients, while outdoor exercise can increase exposure to asthma exacerbation triggers such as cold air, and air pollution^2,6^. On the other hand, studies have demonstrated that engaging in physical activity is associated with better asthma control and^2–4^reduced exercise-induced bronchoconstriction (EIB)^7^. Therefore, children with asthma not only can participate in physical activity, but should be encouraged to do so, as a method to control asthma symptoms and reduce EIB^6,7^. However, many children with asthma do not reach the recommended levels of physical activity, either due to physiological limitations and or habits of physical inactivity^7–9^. Parental or individual anxiety related to EIB may also hinder children reaching adequate physical activity levels, with a vicious cycle of inactivity often established, resulting in both worsened EIB and the loss of other benefits related to physical activity^7,8^.

Generally, exercise during early childhood establishes patterns and defines behaviors and obesity later in life^10^, making childhood a critical period to study the relationship between asthma and physical activity^11^. To date, most of the literature on asthma and physical activity has focused on comparing the physical activity levels of children with and without asthma^2,12^ and has found that people with asthma engage in less physical activity compared to those without^13^. Most previous studies have used self-reported measures for physical activity which are limited by non-response and recall biases, as well as lack of validation^2^. Only few studies have objectively quantified physical activity in this group with the use of accelerometers, even for limited periods of few days, and explored factors affecting physical activity levels among children with asthma^12,14^. Furthermore, although several studies have found that environmental parameters impact physical activity levels of non-asthmatic children^15,16^, we did not identify any studies investigating the impact of environmental parameters such as particulate matter (PM) levels, temperature, and precipitation, on objectively measured physical activity levels of children with asthma. Studying the impact of weather and pollution on physical activity of asthmatic children is critical, given their vulnerability to environmental exposures compared to non-asthmatic children.

This study aimed to prospectively quantify daily physical activity of children with asthma in Cyprus and Crete-Greece using wearable sensors for a period of four months and examine its association with environmental (PM, temperature, and precipitation), anthropogenic, demographic, and clinical parameters.

## Results

In total, 192 children (58% boys, median age 9 years) with asthma participated in the LIFE-MEDEA cohort during the years of 2019 and 2021. Overall, 109 (57%) were recruited in Crete-Greece and 83 (43%) were recruited in Cyprus. Table 1 summarizes their demographic and clinical characteristics. A small subset withdrew from the cohort (*n* = 2) or participated without wearing the smartwatch (*n* = 3). After the exclusion of observation days during which participants did not wear the smartwatch at all and observation days with very low smartwatch wear-time, 186 participants and 7,248 observation days were available for analysis.

**Table 1:** Demographic and clinical characteristics of children with asthma in the LIFE-MEDEA cohort.

*N* = 192 children	*N*	Median^1^	IQR
Age (yrs)	192	9	(8;11)
Gender (boy)	192	111 (57.81)	
BMIz (age-adjusted z-score)	172	0.885	(-0.2;1.84)
Atopy	121	71 (58.68)	
Baseline c-ACT score	167	24	(22;26)
Baseline FEV1% predicted	140	103.2	(93.21;111.64)
Baseline FVC % predicted	134	103.89	(96.77;112.9)
Intervention group^2^	192		
Controls		73 (37.50)	
Outdoor Intervention		53 (27.60)	
Combined Intervention		68 (34.90)	
Country	192		
Cyprus		83 (43.223)	
Crete, Greece		109 (56.77)	
Daily number of steps (total)^3^	7,248	9705.5	(2026;13034)
Daily number of steps (outside house & classroom)	5,150	5948	(3622;8798)
Daily number of steps (at school)	3,599	5012	(3632;6772)
Daily number of steps (at school, outside)	3591	4172	(2758;5928)
Daily number of steps (afternoon)	7,123	4439	(2580;7042)
Daily number of steps (afternoon, outside)	4,221	2308	(839;4671)

IQR interquartile range, BMI body mass index, c-ACT childhood asthma control test, FEV1 forced expiratory volume in one second, FVC forced vital capacity, pp percent predicted.

^1^ Number and percentage N (%) are reported for categorical variables.

^2^ The LIFE-MEDEA study is an intervention study, investigating interventions aimed to reduce desert dust exposure. Participants were assigned to one of three groups: (1) no intervention (2) interventions for outdoor exposure, and (3) interventions for both indoor and outdoor exposure.

^3^ Number of observation days are reported for step data.

### Area weather and atmospheric conditions

Across all observation days, the daily median temperatures were 16.6˚C and 20.9˚C, and the median precent humidity 71.2 (IQR:10.2) and 46.5 (IQR:22) in Crete and Cyprus, respectively (Table S1). Precipitation data were available for 5,674 observation days with Cyprus having 795 (24.6%) and Crete 866 (35.9%) observation days with precipitation. PM_10_ data were available for all 7,248 observation days, and the median daily PM_10_ levels were 28.1 µg/m^3^ (IQR:11.8) in Crete and 33.1 µg/m^3^ (IQR: 12.1) in Cyprus. PM_2.5_ data were available for 3,233 days, all of which were observations from Cyprus where the median daily PM_2.5_ level was 15.6 µg/m^3^ (IQR:7.9).

### Physical activity distributions

**Fig. 1:**
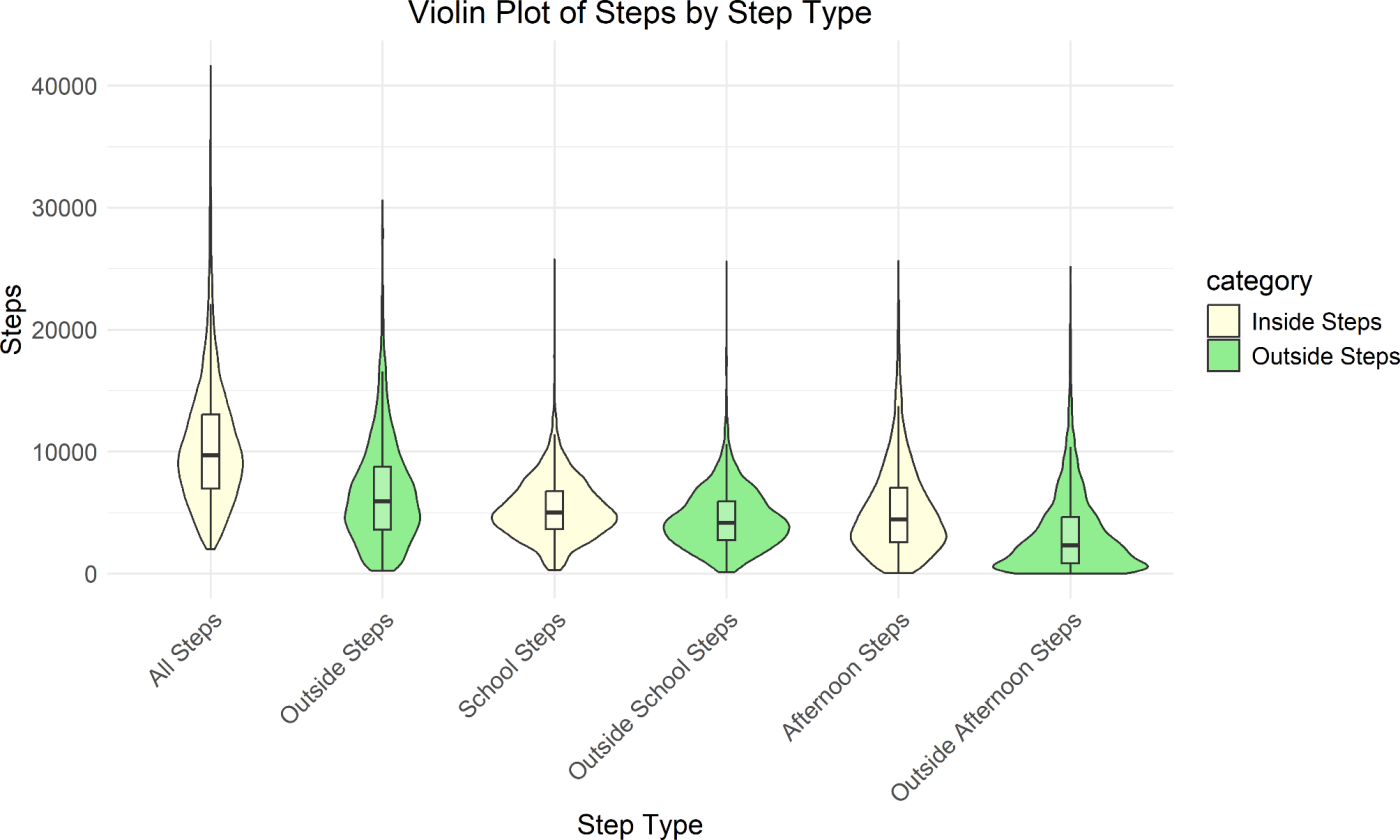
Violin plot of daily steps: Distribution of steps, inside and outside home/classroom, across the whole day and during school and afternoon time periods.

Across all observation days, the median daily steps were 9,706 (IQR: 11,008) (Table 1), while the median daily outside steps were 5,948 (IQR: 5,176). The median daily steps for school and afternoon hours were 5,012 (IQR: 3,140) and 4,439 (IQR: 4,462), respectively. The distributions of daily steps across the different time periods are summarized using violin plots in Fig. 1.

### Effect of environmental parameters on physical activity

In the adjusted mixed effect regression models, we found that precipitation was associated with a decrease of 796 (*β*: -796, 95%CI: -1080, -512, p_value_<0.001) steps per day compared to days without any precipitation (Table 2). We also found that an increase in 10 µg/m^3^ PM_10_ was associated with a decrease of 96 steps per day (*β*: -96, 95%CI: -182, -9, p_value_=0.030). In contrast, changes in PM_2.5_ levels were not found to be significantly associated with physical activity.

**Table 2:** Coefficients for environmental parameters of mixed effects model assessing steps per day (all days, hot days, and cold days).

	Environmental Parameter	Coefficient	95% CI		*p* value
All days	Temperature per ˚C	43	-7	93	0.093
	Rain (binary)	-796	-1080	-512	< 0.001
	PM_10_ per 10 µg/m^3^	-96	-182	-9	0.030
	PM_2.5_ per 10 µg/m^3^	69	-286	423	0.711
Hot days^1^	Temperature per ˚C	-204	-349	-58	0.006
	Rain (binary)	595	-861	2051	0.423
	PM_10_ per 10 µg/m^3^	197	-181	576	0.307
	PM_2.5_ per 10 µg/m^3^	-33	-671	604	0.919
Cold days^2^	Temperature per ˚C	298	129	467	0.001
	Rain (binary)	-1215	-1749	-680	< 0.001
	PM_10_ per 10 µg/m^3^	-199	-328	-71	0.002
	PM_2.5_ per 10 µg/m^3^	281	-755	1317	0.595

CI confidence interval, p-value probability value, PM_10_ particulate matter 10 micrometers and smaller, PM_2.5_ particulate matter 2.5 micrometers and smaller.

^1^Hot days refer to days > 80th percentile temperature for each country.

^2^Cold days refer to days < 20th percentile temperature.

**Fig. 2:**
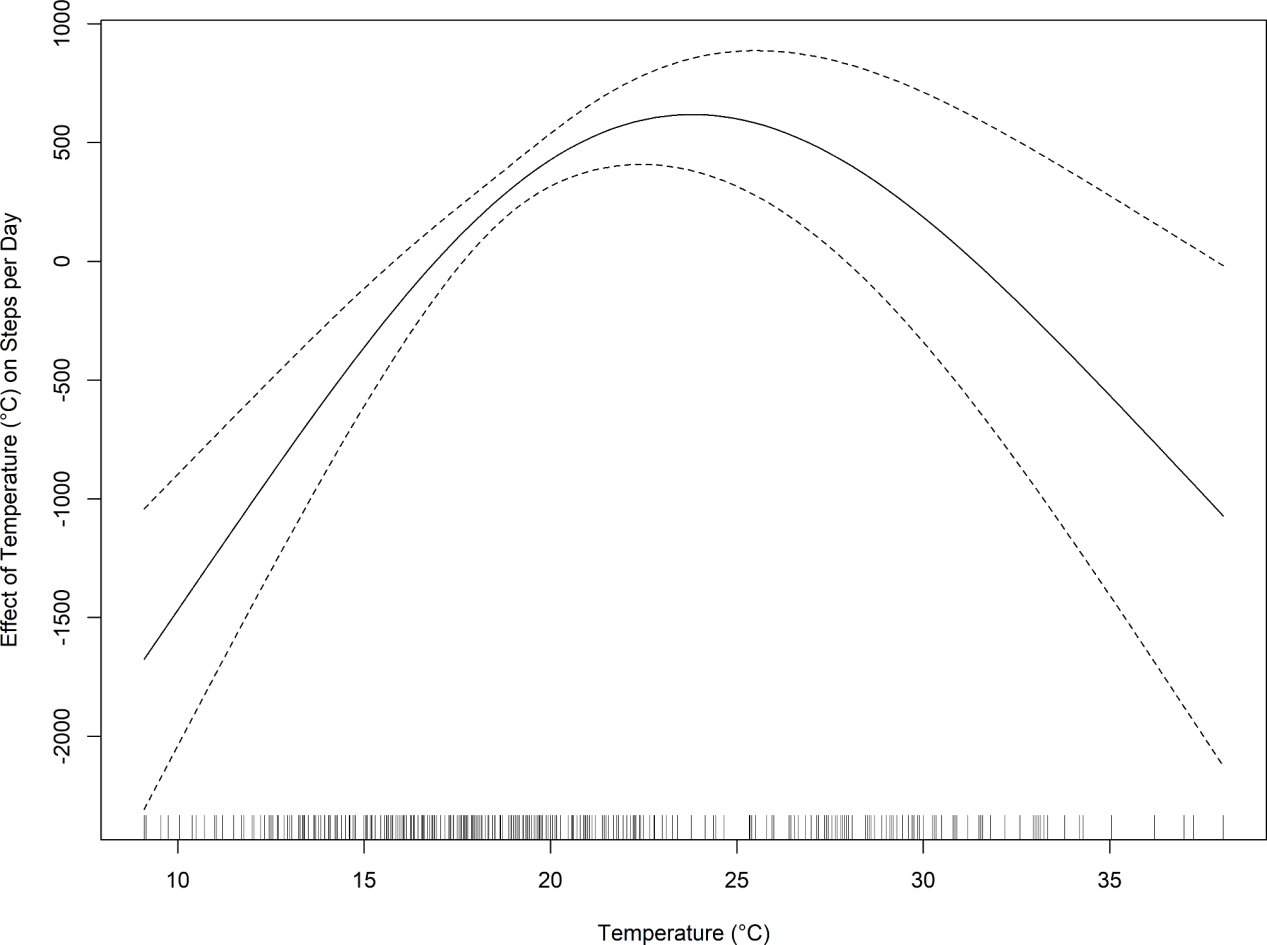
Nonlinear relationship between temperature and daily steps: Inverted U-shaped curve between temperature and daily steps.

Finally, in the main analysis, which employed a linear function to model the relationship between temperature and daily steps, this was not statistically significant (*β*: 43, 95%CI: -7, 93, p_value_ = 0.093 (Table 2). Nevertheless, in post-hoc assessments with a generalized additive mixed model, we demonstrated that temperature modelled as a natural cubic spline with three knots, best fitted the data (BIC: 121,494.1 and AIC: 121,372.8) and an inverted U-shaped curve summarized the nonlinear relationship between temperature and steps (Fig. 2). In post-hoc analyses stratified by hot and cold days, we found a decrease of 204 total daily steps for every increase in degrees Celsius during hot days (β: -204, 95%CI: -349, -58, p_value_ =0.006) and the inverse relationship between temperature and total steps for cold days (β: 298, 95%CI: 129, 467, p_value_=0.001). The effects of rain (β: -1,215, 95%CI: -1,749, -680, p_value_<0.001) and PM_10_ (β: -199, 95%CI: -328, -71, p_value_=0.002) were retained only for cold days (Table 2).

In subgroup analyses restricted to either school or afternoon hours, the observed association between PM_10_ and physical activity remained significant, with every 10 µg/m^3^ increase in PM_10_ being associated with a decrease of 77 steps during both school hours (β: -77, 95%CI: -141,-13, p_value_=0.018) and afternoon hours (β: -77, 95%CI: -145, -10, p_value_=0.024) (Table S2). During school hours only, there was evidence of a positive association between PM_2.5_ and physical activity (p_value_=0.049). Regarding precipitation, the inverse association between rain and physical activity remained significant only during afternoon hours (*β*: -562, 95%CI: -787, -336, p_value_<0.001). In subgroup analyses stratified by cold and hot days, we found that the effect of rain on school hours and afternoon steps was retained for cold days and was more profound in afternoon hours (*β*: -911, 95%CI: -1288, -535, p_value_<0.001) compared to school hours (*β*: -550, 95%CI: -947, -154, p_value_= 0.007). The same was true for PM_10_ during cold days with a negative relationship observed during afternoon hours (*β*: -113 steps per 10 µg/m^3^ PM_10_ increase, 95%CI: -203, -23, p_value_=0.014) as opposed to a non-significant relationship observed during school hours (*β*: -57 steps per 10 µg/m^3^ PM_10_ increase, 95%CI: -161, 47, p_value_=0.281).

In models for outside home and classroom steps the effect of rain remained (*β*: -739, 95%CI: -1061, -417, p_value_<0.001) (Table 4). Stratified analyses revealed that this association was strongest for cold days (*β*: -1268, 95%CI: -1851, -686, p_value_<0.001) (Table S3). As in the main analysis, we found overall a null association between temperature and outside steps per day; however, on cold days we observed an increase in 233 steps per unit increase in degree Celsius (*β*: 233, 95%CI: 41, 425, p_value_<0.017) and on hot days we found a decrease in 322 steps per unit increase in degree Celsius (β: -322, 95%CI: -485, -158, p_value_<0.001).

### Effect of personal and clinical parameters on physical activity

Boys took on average 1,014 (*β*: 1,014, 95%CI: 84.05, 1,944, p_value_ =0.033) more steps per day than girls. In analyses stratified by hot and cold days, we found that the association between gender and physical activity remained consistent with boys demonstrating on average 1,223 and 1,026 more steps per day, compared to girls, on hot and cold days, respectively (p_value_=0.040). There was also evidence of a negative association between BMI z-score with steps per day (*β*: -297, 95%CI: -636, 43, p_value_=0.087); this relationship has being also consistent during hot (*β*: -441, 95%CI:-882,1, p_value_=0.050) and cold days (β: -597, 95%CI:-963,-202, p_value_=0.001) (Table 3).

**Table 3:** Coefficients for clinical and demographic parameters of mixed effects model assessing steps per day (all days, hot days, and cold days).

	Clinical Characteristics	Coefficient	95% CI		*p* value
All days	c-ACT	-4	-54	47	0.891
	Age (yr)	18	-256	292	0.897
	BMIz	-297	-636	43	0.087
	Gender (boy)	1014	84	1944	0.033
	Atopy	455	-657	1567	0.422
	FEV1%	-15	-53	22	0.425
	FVC %	-25	-68	17	0.240
Hot days^1^	c-ACT	-26	-195	143	0.762
	Age (yr)	-57	-402	289	0.748
	BMIz	-441	-882	1	0.050
	Gender (boy)	1223	56	2390	0.040
	Atopy	803	-818	2424	0.332
	FEV_1_%	-6	-62	50	0.831
	FVC %	-22	-87	42	0.495
Cold days^2^	c-ACT	-2	-114	109	0.967
	Age (yr)	35	-249	319	0.808
	BMIz	-597	-963	-202	0.001
	Gender (boy)	1026	49	2004	0.04
	Atopy	731	-374	1836	0.195
	FEV_1_%	4	-35	42	0.858
	FVC %	1	-43	45	0.966

CI confidence interval, p-value probability value, BMI body mass index, c-ACT childhood asthma control test, FEV_1_% forced expiratory volume in one second, FVC % forced vital capacity, percent predicted.

^1^Hot days refer to days > 80th percentile temperature for each country.

^2^Cold days refer to days < 20th percentile temperature.

**Table 4:** Coefficients of mixed effects model assessing outside steps per day.

Parameter	Coefficient	95% CI		*p* value
Environmental				
Temperature (˚C)	19	-38	75	0.516
Rain (binary)	-739	-1061	-416	< 0.001
PM_10_ per 10 µg/m^3^	39	-65	142	0.465
PM_2.5_ per 10 µg/m^3^	45	-36	45	0.826
Clinical & Demographic				
Baseline ACT	11	-46	68	0.708
Age (yr)	53	-171	277	0.641
BMIz	-96	-37	182	0.499
Gender (male)	963	192	1734	0.014
Atopy	98	-680	1	0.805
FEV_1_%	4	-26	34	0.798
FVC %	-9	-44	26	0.618

CI confidence interval, p-value probability value, PM_10_ particulate matter 10 micrometers and smaller, PM_2.5_ particulate matter 2.5 micrometers and smaller, BMI body mass index, c-ACT childhood asthma control test, FEV_1_% forced expiratory volume in one second, percent predicted, FVC % forced vital capacity, percent predicted.

The effect of gender on physical activity was also similar for outside home and classroom steps across all days, with boys taking on average 963 (*β*: 963, 95%CI: 192, 1734, p_value_=0.014) more steps than girls (Table 4). This effect was strongest for hot days (*β*: 1334, 95%CI: 391, 2277, p_value_=0.015) (Table S3). We did not find significant associations between any of the other personal or clinical parameters and outside steps for all, cold, or hot days (Table 4) (Table S3).

## Discussion

In this study, we objectively assessed the impact of environmental and personal parameters on physical activity of children with asthma using wearable sensors. Our findings indicate that among personal parameters, male gender and BMI were positively and negatively associated with total daily steps, respectively. Among environmental parameters, precipitation and PM_10_ were negatively associated with daily steps. The relationship between temperature and daily steps was nonlinear, characterized by an inverted U-shape.

Gender differences in physical activity among children with asthma are consistent with a previous report from Cyprus that also used objective measures of physical activity, albeit for a shorter duration of follow-up and more narrow age range (8–9 years)^14^. Similarly, the systematic review by Vasconcello-Casillo et al. ascertained that the majority of studies that conducted sub-analyses by gender found that boys consistently demonstrate higher levels of physical activity compared to girls^2^. These results mirror the trend among the general population, where from a young age girls are less physically active and have lower levels of cardiorespiratory fitness compared to boys^14,17,18^. In children with asthma, these findings may also reflect gender differences in asthma treatment, where girls are less likely to receive professional counseling or medical treatment for asthma compared to boys leading to decreased physical activity^14^. Furthermore, the design and facilities of many playgrounds may be less appealing towards activities often preferred by girls, thereby contributing to this disparity^18^.

Our results also corroborated pre-existing findings of an inverse relationship of BMI with physical activity. Characteristically, a study from the same region (Greece) assessing pedometer-measured physical activity in 9- to 14-year-old non-asthmatic children found that overweight and obese children were significantly less physically active when compared to normal weight children^17^. Similar findings were reported for adults with asthma^19^. In addition, it has been demonstrated that physical activity mediates the relationship between asthma and high BMI in children and adolescents^20^. Nevertheless, the temporal relationship between asthma, BMI, and physical activity remains unclear. Possible pathways, including negative self-perception or parental and/or personal anxiety related to EIB and other symptoms secondary to physical exertion, lead from asthma to sedentary lifestyle and higher BMI^7–9^. On the other hand, withstanding inconsistent data, obesity is considered a risk factor for the development and worsening of asthma;^21,22^ based on this notion, several researchers have examined the aimed to demonstrate the benefits of weight intervention studies with mixed results, especially in children^23,24^.

Our study found that higher PM_10_ concentrations decreased daily steps. This result is in line with a previous analysis from this cohort that focused on desert dust storm days and demonstrated that, in general, children with asthma reduced their steps outside home and classroom during desert dust storm days compared to non-desert dust storm days. Especially for children with asthma that received intervention with early warnings and behavioral recommendations to reduce outdoor physical activity during desert dust days, the effect was much more profound. In more detail, the change in daily steps performed outside classrooms and homes during desert dust days, was − 495 steps (pvalue = 0.350) in asthmatic children not receiving any intervention and − 1040 steps (pvalue = 0.003) in asthmatic receiving the intervention^25^. Similar to our findings, a prospective study by Yu and Zhang of non-asthmatic Chinese children, using objectively measured physical activity, demonstrated that PM_2.5_ and more so PM_10_ were associated with a decrease in physical activity^15^. This decrease in physical activity could be due to a reduction in physical activity following media air pollution alerts^15^ or due to behavioral adaptations. As air pollution is a known trigger for bronchoconstriction, children with asthma and their care takers may refrain from going outside during periods of high air pollution^2,6^. This pathway aligns with our finding that PM_10_ had the greatest effect on cold days, another trigger for bronchoconstriction, because there may extra care to avoid these co-exposures^6^.

The association between temperature and physical activity in children with asthma is described for the first time in this study. We report an inverted U-shape relationship, which largely aligns with findings from studies involving non-asthmatic children^26^. Children with asthma may be more sensitive to temperature changes because colder air is a trigger for bronchoconstriction and hot and humid air makes it more difficult to breathe^6,27^. Not surprisingly, caregivers often monitor weather conditions in advance and may prefer indoor and more sedentary activities to minimize asthma exacerbation risk on days with unfavorable weather^28,29^. Furthermore, aligning with studies of non-asthmatic children, in our study precipitation was negatively associated with asthmatic children’s daily steps^26^. Notably, our finding that this impact was strongest for afternoon hours suggests that parental anxiety may predominantly drive this association^7^. The absence of a negative association between precipitation and daily steps during hot days may be explained by the potential cooling effect of cloud cover that may encourage physical activity on hot days.

Engaging in physical activity is beneficial for children with asthma and can help build healthy habits that continue into adulthood;^10^ however, many children with asthma are not reaching the recommended physical activity levels^6,7^. We found that precipitation, extreme temperatures, air pollution, and personal characteristics such as gender and BMI, in conjunction with personal and parental behaviors may create barriers for children with asthma to engage in physical activity.

In terms of weather and air pollution, planning for days with increased precipitation, extreme temperatures -both of which are expected to increase with ongoing climate change- and high air pollution can help children meet physical activity recommendations^26^. This may include creating indoor spaces for physical activity, such as gymnasiums, organizing indoor physical activities, and planning physical activities to align with seasonal weather patterns^26,30^. In^18^addition, special care should be given by local and school authorities to design playground facilities and fund playground activities that appeal to girls^18^. Further, asthmatic girls’ engagement in physical activities should be supported by their healthcare providers and caretakers, including addressing societal norms and stereotypes related to appropriate kinds of physical activities, body image perception, and conceptions around sweating, which may create poise barriers for girls engaging in physical activity^14^. Other considerations for the design of urban environments and school infrastructure that can further promote physical activity among asthmatic children include ensuring high air quality in both indoor and outdoor areas used for physical activity. In indoor areas, this can be achieved by appropriate ventilation systems^31^ or use of indoor air purifiers^32,33^. In the outdoor setting, this can be achieved with the efficient use of green infrastructure, such as the introduction of hedges of appropriate barrier height, porosity and length between traffic and areas of outdoor physical activity, promoting the creation of urban “green oases” that are characterized by low ventilation and absence of any internal air pollution sources^34^. Furthermore, as extreme high temperatures are expected to increase in many areas of the world in the context of climate change, shading interventions including enhanced green canopy or shade sails in urban parks are needed to improve thermal comfort, which may also promote physical activity during the warmer months^35,36^. Beyond the likely direct effects of environmental parameters on physical activity in this sensitive population group, it is likely that some of these associations may be mediated by self/parental fear and anxiety around asthma symptoms and EIB^7,8^. To address these anxieties, more resources should be provided to families of children with asthma that explain the importance of physical activity, the ways to safely engage in physical activity, and how to control asthma^37^. Personalized treatment plans could be created that include physical activity and advise parents and children on what conditions are safe or risky for children with asthma^38^.

To the best of our knowledge, our study is the first study to assess the impact of environmental and other parameters on physical activity among children with asthma in the Eastern Mediterranean region. The major strength of this study is the use of pedometer and geolocation data from wearable sensors to objectively assess physical activity, as opposed to studies based on subjective recall of physical activity, which may be biased. Furthermore, unlike previous studies using objectively measured physical activity data, our study acquired data from a relatively long follow-up period (4 months) and a large number of children with asthma (*n* = 186).

Nevertheless, our study has some weaknesses. Firstly, a comparison control group of healthy children was not included in the study, and it is unclear if the effects of environmental, demographic and clinical parameters on physical activity would have been similar in healthy children as well. In addition, the study population included mostly children with well controlled asthma and as a result the estimates of this study may be different in children with more severe asthma and reduced lung function. Previous studies have demonstrated that physical fitness is lower in children and adolescents with severe asthma compared to those with mild asthma^39^ and it is possible that the negative associations between air pollution and unfavorable weather conditions may be even more profound among children with severe asthma. Moreover, the relationship between asthma and determinants of physical activity in children may be influenced by socio-economic status^40^ and other relevant parameters including race^41^, housing conditions^42^ and neighborhood factors^43^. Similarly, risk perception regarding air pollution^44^ as well as perceived competence and attitude toward physical activity^9^ have also been shown to affect physical activity and aerobic fitness among children with asthma. Therefore, additional studies in severe asthma groups and in other geographical regions are required to explore further this relationship.

Furthermore, the smartwatch, used for data collection, needed to be recharged at night; therefore, night-hour activity data is missing. However, children are not expected to be active during the night. Future studies utilizing wearable sensors could consider investigating the sleeping patterns and nighttime activity of children with asthma, who often have problems sleeping^45^. Likewise, smartwatches may inaccurately represent cycling and walking upstairs and cannot be worn during swimming. As such, more detailed assessments of different types of physical activities were not possible. Lastly, the accuracy of GPS signal is known to be frequently compromised by signal loss in and around buildings; this may have introduced uncertainty in our assessment of inside and outside steps. To account for this uncertainty, we used a 100 m radius “buffer” around a participant’s home or classroom and relied on a GPS data filling algorithm, as suggested in previous studies^46^.

## Conclusions

Among children with asthma, female gender, BMI, precipitation and PM_10_ are negatively associated with physical activity, while the relationship with temperature is characterized by an inverted U-shaped. These results highlight the role of different parameters on the complex relationship between asthma and physical activity and can further inform the design of interventions to encourage physical activity among children with asthma.

## Materials and methods

### Setting and study population

Cyprus and Crete-Greece are large islands located in the Eastern Mediterranean basin and are characterized by a subtropical climate with dry hot summers, mild rainy winters, and short autumn (October) and spring (April to May) periods of rapid weather change^47^ and mild temperatures^48^.

The design of the LIFE-MEDEA asthma panel study has been described in detail previously^49^. In brief, the study enrolled children aged 6 to 11 with mild to moderate persistent asthma from primary schools in Cyprus and Crete between 2019 and 2021. A new cohort was enrolled each year, and the children were followed up from February through May. For this analysis we excluded data from 2020 due to changes in personal behavior and asthma morbidity in response to the COVID-19 pandemic and resulting lockdown measures^50^.

To be included in this study children needed to have a physician diagnosis of asthma and meet additional inclusion criteria, described in detail in Supplementary Material. Following recruitment, participants were randomized into three intervention groups for exposure reduction to air pollution during the desert dust season at the two sites. These were the control group (no intervention), the outdoor intervention group (received early warnings and recommendations to stay indoors and limit outdoor physical activity during desert dust days) and the combined intervention group (also received early warnings and recommendations during desert dust days as well as had installed indoor air purifiers in houses and classrooms). During desert dust days, there was an overall reduction in physical activity outside home and classroom of children with asthma, but this effect was significantly more pronounced in the intervention groups receiving the early warnings and recommendations. Detailed information regarding the impact of these exposure reduction interventions on health outcomes among children with asthma is available elsewhere^51^ while information on ethics and informed consent is available in Supplementary Material. Briefly, in Cyprus, study approvals were obtained from the Cyprus National Bioethics Committee (EEBK EΠ 2017.01.141), the Data Protection Commissioner (No. 3.28.223) and the Ministry of Education (No 7.15.01.23.5). In Greece, approvals were obtained from the Scientific Committee (25/04/2018, No: 1748) and the Governing Board of the University General Hospital of Heraklion (25/22/08/2018). All research described was performed in accordance with relevant national guidelines and in accordance with the Declaration of Helsinki. Guardians of all participants provided written informed consent for their participation.

### Physical activity and GPS tracking

Physical activity and GPS data of participating children were continuously assessed through wearable technology, as previously described^25^. In brief, upon recruitment the geographic coordinates of children’s home and classroom locations were entered to the MEDnea ^®^ Health-Hub, a bi-directional, patient-centered web-based platform, and each child was given an EMBRACE™ smartwatch that was linked to their participant ID. The EMBRACE™ smartwatch is equipped with multiple sensors to continuously collect global positioning system (GPS) and activity data, as well as software to synchronize the transfer of data to the MEDnea ^®^ Health-Hub. Data were collected in 5-minute intervals and uploaded automatically when participants’ smartwatches connected to their home Wi-Fi network. Participants were asked to wear the smartwatch continuously for seven days a week, excluding time when sleeping or swimming.

Children’s total “daily steps” (24 h period) and “daily outside” home and classroom steps were recorded. “Outside” was defined as outside of a 100-m radius from participants’ residences and classrooms. We used a 100-m radius to account for limitations in the signal accuracy of commercially available GPS receivers and because participants were in urban areas/indoor environments, where buildings may bounce or block GPS signals. Observation days during which participants did not wear the smartwatch at all (identified by absence of heart rate measurements) or observation days characterized by low smartwatch wear time (< 6 h of data collected) were a-priori excluded from this analysis to ensure data quality as described previously^25,52^.

### Personal and clinical measurements

Gender, age, height, weight, lung function, scores on childhood Asthma Control Tests (cACT), information on asthma medication use, and information on unscheduled asthma-related clinician visits were recorded at baseline. Throughout follow-up, repeated assessments of cACT scores, lung function (FEV1% predicted and FVC% predicted) and FeNO were conducted. We also collected information on asthma medications use/clinician visits. Atopic sensitization was assessed at the end of follow-up by skin prick testing to 14 common airborne allergens^51^. Additional information on clinical measurements is available in Supplementary Material.

### Environmental measurements

We obtained daily mean values of temperature, relative humidity, and precipitation from the urban meteorological station closest to participants’ residences while we obtained daily mean values of PM_2.5_ and PM_10_ for Nicosia, Cyprus and Heraklion, Crete from the corresponding urban air quality monitoring station. Additional details on data sources are presented in Supplementary Material.

### Statistical analysis

Adjusted mixed effect regression models were used to examine the associations of environmental factors (temperature (˚C), precipitation (mm), PM_10_ (µg/m^3^), and PM_2.5_(µg/m^3^) (Eq. 1, Supplementary Material) and personal characteristics (age (years), gender, age-adjusted BMI z-scores, asthma symptom control, atopy status, and lung function (FEV_1_% predicted, FVC% predicted) (Eq. 2, Supplementary Material) on physical activity quantified as steps per day (all-day steps, school-hour steps, and afternoon steps). Mixed models were employed to account for repeated observations for each participant, and models were adjusted for seasonality, country, day of the week as well as for intervention group to control for potential confounding from the implementation of behavioral recommendations during dust-days. Public holidays were excluded from the analyses.

In the main analysis, the association between exposure variables and steps per day was modelled using a linear function. However, in post-hoc assessments of the model-fit we found that for temperature, a nonlinear spline model was more appropriate. We examined the fit of cubic spline models with 2, 3, 4, 5, and 6 knots, and chose the final model by comparing Bayesian information criterion (BIC) values. We further investigated the relationship between unseasonal temperatures and physical activity by repeating the analysis for warm (≥ 80th percentile) and cold days (≤ 20th percentile). All statistical analyses were carried out using STATA (Version 17, StataCorp, College Station, TX) and mixed effect regression models were developed using the *xtmixed* command, which uses Maximum Likelihood Estimation to calculate fixed and random effects, even if some predictor data were missing in certain observation days (rows). With this approach the likelihood function for the other predictors and the outcome variable (steps) was still calculated using the remaining available data, thus maximizing data usage. Statistical significance was set at *p* < 0.05.

## Electronic supplementary material

Below is the link to the electronic supplementary material.

Supplementary Material 1

## Data Availability

The data that support the findings of this study are not openly available due to reasons of sensitivity and are available from the corresponding author upon reasonable request. Data are located in controlled access data storage at Univeristy of Cyprus.

## Abbreviations

EIB: Exercise-induced bronchoconstriction
PM: Particulate matter; GPS: Global Positioning system
cACT: childhood Asthma Control Test
FEV1: Forced expiratory volume in 1 s
FVC: Forced Vital Capacity
BMI: Body Mass Index
IQR: Interquartile Range
BIC: Bayesian Information Criterion
