# Supplementary material for: Association of environmental, demographic and clinical parameters with physical activity in children with asthma
Source: Sci Rep. 2025 Jan 22;15:2886. doi: 10.1038/s41598-025-87426-5 (PMC11754804; doi:10.1038/s41598-025-87426-5)
Supplement: Supplementary file 1 — Supplementary Material 1 [file 41598_2025_87426_MOESM1_ESM.docx]

**Supplementary Material for manuscript**

“Association of environmental, demographic and clinical parameters with physical activity in children with asthma”

**Supplementary information (text):**

**Additional information on Study Population**

To be included in this study children needed to have a physician diagnosis of asthma and meet at least one of the following criteria: 1) daily intake of preventative asthma medication, 2) report of wheezing episodes in the past year, and 3) report of unscheduled medical visits for asthma in the past year. Children were excluded if they had lung diseases other than asthma, cardiovascular disease, or did not live in the same household for at least 5 days a week.

**Information on ethics and informed consent**

In Cyprus, the study was approved by the Cyprus National Bioethics Committee (EEBK EΠ 2017.01.141), the Data Protection Commissioner (No. 3.28.223), and the Ministry of Education (No 7.15.01.23.5). In Greece, the study was approved by the Scientific Committee (25/04/2018, No: 1748) and the Governing Board of the University General Hospital of Heraklion (25/22/08/2018). Guardians of all participants provided written, informed consent for their participation.

**Additional information on environmental measurements**

We obtained daily mean values of temperature, relative humidity, and precipitation from the urban meteorological station closest to participants’ residences (for Cyprus: Department of Meteorology, Cyprus Ministry of Agriculture, Rural Development and Environment and for Crete: Hellenic National Meteorological Service). Similarly, we obtained daily mean values of PM_2.5_ and PM_10_ for Nicosia, Cyprus from the Air Quality Section, Department of Labour Inspection, Ministry of Labour and Social Insurance and for Heraklion, Greece from the Environmental Chemical Processes Laboratory of the Department of Chemistry of the University of Crete.

**Additional information on clinical measurements**

Body mass index (BMI) (kg/m^2^) was expressed as age- and gender- specific z-scores based on the WHO 2007 References for School-age Children and Adolescents (5 to 19 years) using the “anthroplus” package in R. The Greek version of the c-ACT questionnaire was administered monthly by telephone interviews (c-ACT, license number: QM044906) and assessed asthma symptom control in the past four weeks. Lung function and FeNO were assessed at baseline, at the midpoint and at the end of the follow-up period. Lung function was assessed using a portable spirometer (In2itive Spirometer, Vitalograph Ltd., United Kingdom) and fractional exhaled nitric oxide, by a portable nitric oxide analyzer (FeNO- NIOX VERO Circassia, United Kingdom).

**Additional information on statistical analysis**

Adjusted mixed effect models (equations)

Equation 1: ${Steps}_{ij}= \beta_{0}+\beta_{1}{Temperature}_{ij}+\beta_{2}{Precipitation}_{ij}+\beta_{3}{PM}_{ij}+\beta_{3}{Seasonality}_{ij}+\beta_{4}{Group}_{i}+\beta_{5}{Country}_{i}+\beta_{6}{dow}_{ij}+u_{i}+e_{ij}$

Equation 2: ${Steps}_{ij}= \beta_{0}+\beta_{1}{Age}_{ij}+\beta_{2}{Gender}_{ij}+\beta_{3}{BMIz}_{ij}+\beta_{4}{ACT}_{ij}+\beta_{5}{Atopy}_{i}+\beta_{6}{{LungFunction}_{ij}{+ \beta}_{7}{Seasonality}_{ij}+\beta}_{8}{Group}_{i}+\beta_{9}{Country}_{i}+\beta_{10}{dow}_{ij}+u_{i}+e_{ij}$

Where Steps_ij_ is the change in steps for participant i and day j, while the fixed effect portion of the model includes the population intercept and slope coefficients for each independent variable. The term u_i_ denotes the random effect (intercept) for each participant and e_ij_ the error term for participant i and day j.

**Figure S1:** **Violin plot of daily steps stratified by gender:** Distribution of steps across the whole day and during school and afternoon time periods, stratified by gender.


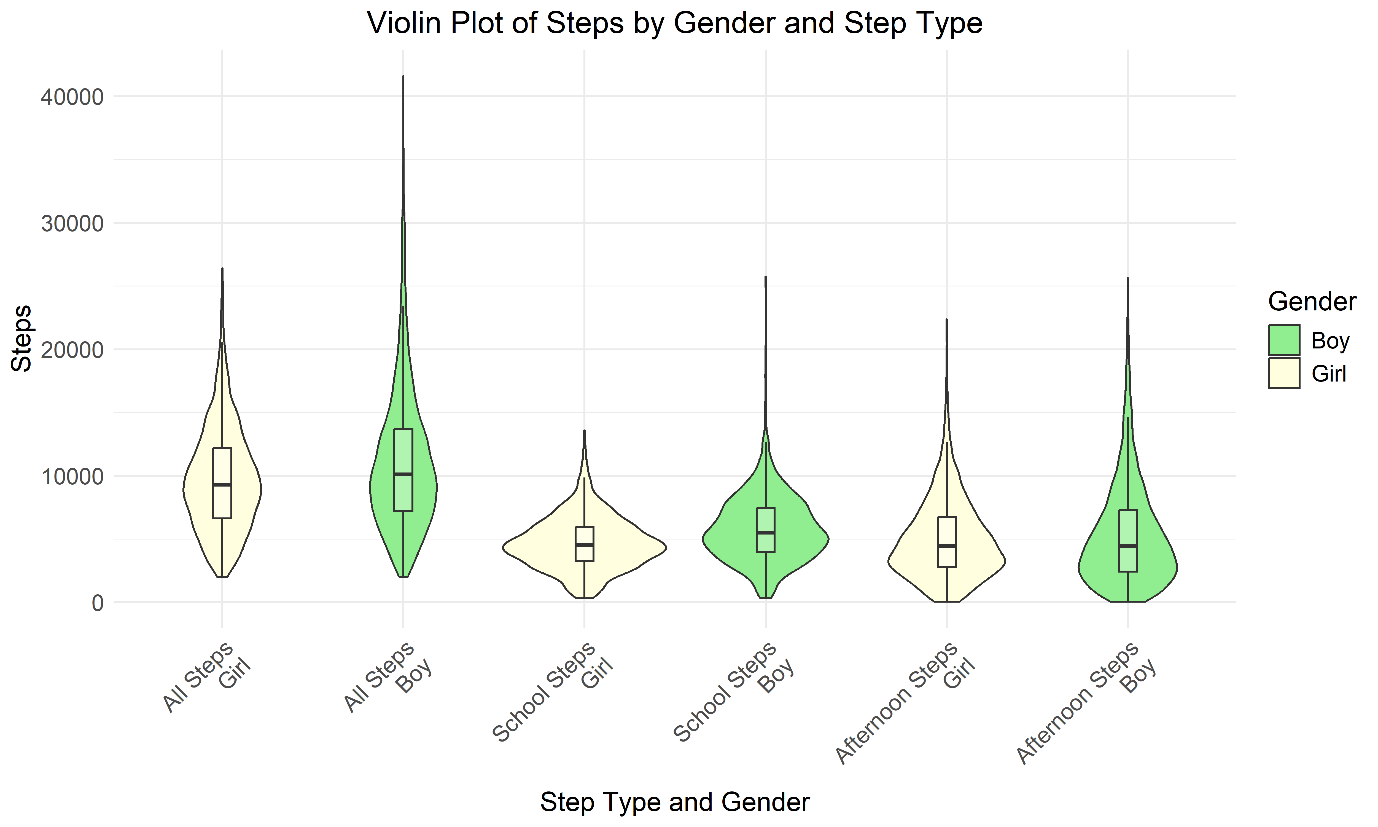


| **TABLE S1:** Area weather conditions by country | | | | |
| --- | --- | --- | --- | --- |
|  | **Cyprus** | | **Crete-Greece** | |
|  | **Median*** | **IQR** | **Median*** | **IQR** |
| **Temperature (˚C)** | 20.9 | 11.8 | 16.6 | 5.1 |
| **Precipitation days** | 795 (24.6) |  | 866 (35.9) |  |
| **Precipitation (mm)**** | 1.7 | 5.2 | 0.7 | 3.2 |
| **Percent humidity** | 46.5 | 22.0 | 71.2 | 10.2 |
| **PM_10_ μg/m^3^** | 33.1 | 12.1 | 28.1 | 11.8 |
| **PM_2.5_ μg/m^3^** | 15.6 | 7.9 | - | - |
| Abbreviations: IQR interquartile range, PM10 particulate matter 10 micrometers and smaller, PM2.5 particulate matter 2.5 micrometers and smaller | | | | |
| * Number and percentage N (%) are reported for categorical variables | | |  |  |
| ** Median and IQR are calculated for days with precipitation | | |  |  |

| **TABLE S2:** Coefficients for environmental parameters of mixed effects model assessing steps during school hours and the afternoon (all days, hot days, and cold days) | | | | | | | | | | |
| --- | --- | --- | --- | --- | --- | --- | --- | --- | --- | --- |
|  | | **School hours** | | | | **Afternoon hours** | | | | |
|  | **Environmental Parameter** | **Coefficient** | **95% CI** | | **p value** | **Coefficient** | **95% CI** | | **p value** |  |
| **All days** | **Temperature (˚C)** | -3 | -38 | 33 | 0.884 | 24.99 | -15 | 65 | 0.215 |  |
|  | **Rain (binary)** | -75 | -282 | 132 | 0.476 | -561.74 | -787 | -336 | <0.001 |  |
|  | **PM_10_ per 10μg/m^3^** | -77 | -141 | -13 | 0.018 | -77.43 | -145 | -10 | 0.024 |  |
|  | **PM_2.5_ per 10μg/m^3^** | 258 | 2 | 514 | 0.049 | -120.66 | -416 | 175 | 0.424 |  |
| **Hot Days^1^** | **Temperature (˚C)** | -29 | -143 | 84 | 0.611 | -47.9 | -172 | 76 | 0.449 |  |
|  | **Rain (binary)** | -89 | -1459 | 1280 | 0.898 | 627.2 | -609 | 1864 | 0.320 |  |
|  | **PM_10_ per 10μg/m^3^** | -13 | -272 | 246 | 0.92 | -175.50 | -500 | 149 | 0.289 |  |
|  | **PM_2.5_ per 10μg/m^3^** | 90 | -378 | 557 | 0.707 | -87.21 | -636 | 462 | 0.756 |  |
| **Cold Days^2^** | **Temperature (˚C)** | -43 | -179 | 93 | 0.535 | 178.5 | 60 | 297 | 0.003 |  |
|  | **Rain (binary)** | -550 | -947 | -154 | 0.007 | -911.4 | -1288 | -535 | <0.001 |  |
|  | **PM_10_ per 10μg/m^3^** | -57 | -161 | 47 | 0.281 | -112.67 | -203 | -23 | 0.014 |  |
|  | **PM_2.5_ per 10μg/m^3^** | 125 | -1120 | 1369 | 0.844 | 167.80 | -611 | 967 | 0.673 |  |
| Abbreviations: CI confidence interval, p-value probability value, PM_10_ particulate matter 10 micrometers and smaller, PM_2.5_ particulate matter 2.5 micrometers and smaller.  ^1^Hot days refer to days >80th percentile temperature for each country.  ^2^Cold days refer to days <20th percentile temperature. | | | | | | | | | | |

| **TABLE S3:** Coefficients of mixed effects model assessing outside steps per day for hot and cold days | | | | | | | | | | |
| --- | --- | --- | --- | --- | --- | --- | --- | --- | --- | --- |
|  | **Parameter** | **Coefficient** | | **95% CI** | | | | | **p value** | |
| **Hot days^1^** | *Environmental* |  | |  | |  | | |  | |
|  | **Temperature (˚C)** | -322 | -485 | | | | | -158 | | <0.001 |
|  | **Rain (binary)** | -817 | -2553 | | | | | 919 | | 0.356 |
|  | **PM_10_ per 10μg/m^3^** | 305 | -129 | | | | | 740 | | 0.169 |
|  | **PM_2.5_ per 10μg/m^3^** | -119 | -870 | | | | | 632 | | 0.757 |
|  | *Clinical & Demographic* |  | | |  | |  | | |  |
|  | **ACT** | -2 | -148 | | | | | 144 | | 0.980 |
|  | **Age (yr)** | -32 | -320 | | | | | 255 | | 0.825 |
|  | **BMIz** | -165 | -527 | | | | | 197 | | 0.371 |
|  | **Gender (male)** | 1334 | 391 | | | | | 2277 | | 0.015 |
|  | **Atopy** | 713 | -636 | | | | | 2063 | | 0.300 |
|  | **FEV_1_ %** | -12 | -57 | | | | | 33 | | 0.598 |
|  | **FVC %** | -14 | -67 | | | | | 40 | | 0.610 |
| **Cold days^2^** | *Environmental* |  | | |  | |  | | |  |
|  | **Temperature (˚C)** | 233 | 41 | | | | | 425 | | 0.017 |
|  | **Rain (binary)** | -1268 | -1851 | | | | | -686 | | <0.001 |
|  | **PM_10_ per 10μg/m^3^** | -27 | -183 | | | | | 129 | | 0.734 |
|  | **PM_2.5_ per 10μg/m^3^** | 67 | -1120 | | | | | 1254 | | 0.912 |
|  | *Clinical & Demographic* |  | | |  | |  | | |  |
|  | **ACT** | 3 | -95 | | | | | 101 | | 0.954 |
|  | **Age (yr)** | 54 | -170 | | | | | 278 | | 0.638 |
|  | **BMIz** | -197 | -488 | | | | | 95 | | 0.186 |
|  | **Gender (male)** | 619 | -139 | | | | | 1376 | | 0.109 |
|  | **Atopy** | 98 | -680 | | | | | 877 | | 0.805 |
|  | **FEV_1_ %** | 4 | -26 | | | | | 34 | | 0.798 |
|  | **FVC %** | -9 | -44 | | | | | 26 | | 0.618 |
